# Improved Image Fusion Method Based on NSCT and Accelerated NMF

**DOI:** 10.3390/s120505872

**Published:** 2012-05-07

**Authors:** Juan Wang, Siyu Lai, Mingdong Li

**Affiliations:** 1 College of Computer Science, China West Normal University, 1 Shida Road, Nanchong 637002, China; E-Mails: wjuan0712@126.com (J.W.); mdong_li@163.com (M.L.); 2 Department of Medical Imaging, North Sichuan Medical College, 234 Fu Jiang Road, Nanchong 637000, China

**Keywords:** image fusion, non-subsampled contourlet transform, nonnegative matrix factorization, neighborhood homogeneous measurement

## Abstract

In order to improve algorithm efficiency and performance, a technique for image fusion based on the Non-subsampled Contourlet Transform (NSCT) domain and an Accelerated Non-negative Matrix Factorization (ANMF)-based algorithm is proposed in this paper. Firstly, the registered source images are decomposed in multi-scale and multi-direction using the NSCT method. Then, the ANMF algorithm is executed on low-frequency sub-images to get the low-pass coefficients. The low frequency fused image can be generated faster in that the update rules for *W* and *H* are optimized and less iterations are needed. In addition, the Neighborhood Homogeneous Measurement (NHM) rule is performed on the high-frequency part to achieve the band-pass coefficients. Finally, the ultimate fused image is obtained by integrating all sub-images with the inverse NSCT. The simulated experiments prove that our method indeed promotes performance when compared to PCA, NSCT-based, NMF-based and weighted NMF-based algorithms.

## 1. Introduction

Image fusion is an effective technology that synthesizes data from multiple sources and reduces uncertainty, which is beneficial to human and machine vision. In the past decades, it has been adopted in a variety of fields, including automatic target recognition, computer vision, remote sensing, robotics, complex intelligent manufacturing, medical image processing, and military purposes. Reference [1] proposed a framework for the field of image fusion. The fusion process is performed at different levels of the information representation, which is sorted in ascending order of abstraction: pixel, feature, and decision levels. Of these, pixel-level fusion has been broadly studied and applied for it is the foundation of other two levels.

Pixel-level image fusion consists of two parts: space domain and frequency domain. The classic algorithms in the frequency domain include Intensity Hue Saturation (IHS) [2], Principal Component Analysis (PCA) [3], pyramid [4,5], wavelet [6,7], wavelet packet [8], Dual Tree Complex Wavelet Transform (DT-CWT) [9,10], curvelet [11,12], contourlet [13,14], and Non-subsampled Contourlet Transform (NSCT) [15], *etc.*

Until recently, the multi-resolution decomposition based algorithms have been widely used in the multi-source image fusion field, and effectively overcome spectrum distortion. Wavelet transformation provides great time-frequency analytical features and is the focus of multi-source image fusion. NSWT is made up of the tensor product of two one-dimension wavelets, solving the shift-invariant lacking problem that the traditional wavelets cannot do. Being lacking in anisotropy, NSWT fails to express direction-distinguished texture and edges sparsely. In 2002, Do and Vetteri proposed a flexible contourlet transform method that may efficiently detect the geometric structure of images attributed to their properties of multi-resolution, local and directionality [13], but the spectrum aliasing phenomenon occurs posed by unfavorable smoothness of the basis function. Cunha *et al.* put forward the NSCT method [15] in 2006; improvements have been made in solving contourlet limitations, and it was an ultra-perfect transformation with attributes of shift-invariance, multi-scale and multi-directionality [16].

Non-Negative Matrix Factorization (NMF) is a relatively new matrix analysis method [17] presented by Lee and Seung in 1999, and has been proven to converge to its local minimum in 2000 [18]. It has been successfully adopted in a variety of applications, including image analysis [19,20], text clustering [21], speech processing [22], pattern recognition [23–25], and so on. Unfortunately, some NMF-involved works are time consuming. In order to reduce time costs, an improved NMF algorithm has been introduced in this paper. Our improved NMF algorithm is applied to fuse the low-frequency information in he NSCT domain, while the fusion of high-frequency details can be realized by adopting the Neighborhood Homogeneous Measurement (NHM) technique used in reference [26]. The experimental results demonstrate that the proposed fusion method can effectively extract useful information from source images and inject it into the final fused one which has better visual effects, and the running of the algorithm takes less CPU time compared with the algorithms proposed in [27] and [18].

The remainder of this paper is organized as follows: we introduce NSCT in Section 2. This is followed by a brief discussion on how NMF is constructed, and how we improve it. Section 4 presents the whole framework of the fusion algorithm. Section 5 shows experimental results for image fusion using the proposed technique, as well as the discussion and comparisons with other typical methods. Finally, the last Section concludes with a discussion of our and future works.

## 2. Non-Subsampled Contourlet Transform (NSCT)

NSCT is proposed on the grounds of contourlet conception [13], which discards the sampling step during the image decomposition and reconstruction stages. Furthermore, NSCT presents the features of shift-invariance, multi-resolution and multi-dimensionality for image presentation by using a non-sampled filter bank iteratively.

**Figure 1.**
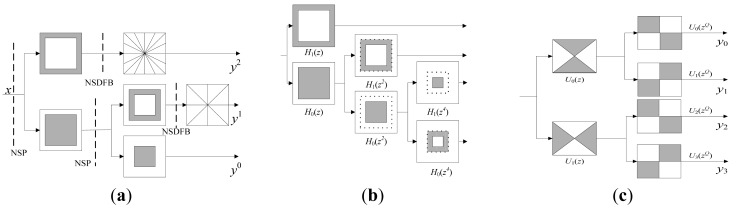
Diagram of NSCT, NSP and NSDFB. (**a**) NSCT filter bands; (**b**) Three-levels NSP; (**c**) Decomposition of NSDFB.

The structure of NSCT consists of two parts, as shown in Figure 1(a): Non-Subsampled Pyramid (NSP) and Non-Subsampled Directional Filter Banks (NSDFB) [15]. NSP, a multi-scale decomposed structure, is a dual-channel non-sampled filter that is developed from the àtrous algorithm. It does not contain subsampled processes. Figure 1(b) shows the framework of NSP, for each decomposition of next level, the filter *H (z)* is firstly sampled an using upper-two sampling method, the sampling matrix is *D* = (2, 0; 0, 2). Then, low-frequency components derived from the last level are decomposed iteratively just as its predecessor did. As a result, a tree-like structure that enables multi-scale decomposition is achieved. NSDFB is constructed based on the fan-out DFB presented by Bamberger and Smith [28]. It does not include both the super-sampling and sub-sampling steps, but relies on sampling the relative filters in DFB by treating *D* = (1, 1; 1, −1), which is illustrated in Figure 1(c). If we conduct *L* levels of directional decomposition on a sub-image that decomposed by NSP in a certain scale, then 2*^L^* number of band-pass sub-images, the same size to original one, are available. Thus, one low-pass sub-image and $\overset{L}{\underset{j=1}{\text{∑}}}2^{l}j$ band-pass directional sub-images are generated by carrying out *L* levels of NSCT decomposition.

## 3. Improved Nonnegative Matrix Factorization

### 3.1. Nonnegative Matrix Factorization (NMF)

NMF is a recently developed matrix analysis algorithm [17,18], which can not only describe low-dimensional intrinsic structures in high-dimensional space, but achieves linear representation for original sample data by imposing non-negativity constraints on its bases and coefficients. It makes all the components non-negative (*i.e.*, pure additive description) after being decomposed, as well as realizes the non-linear dimension reduction. NMF is defined as:

Conduct *N* times of investigation on a *M*-dimensional stochastic vector *v*, then record these data as *v_j_, j* = 1,2,…, *N*, let *V* = [*V*_•1_, *V*_•2_, *V*_•_*_N_*], where *V_•j_* = *v_j_, j* = 1,2,…, *N*. NMF is required to find a non-negative *M* × *L* base matrix *W* = [*W*_•1_, *W*_•2_,…, *W*_•_*_N_*] and a *L* × *N* coefficient factor *H* = [*H*_•1_, *H*_•2_,…, *H*_•_*_N_*], so that *V* ≈ *WH* [17]. The equation can also be wrote in a more intuitive form of that $V_{.j}\approx \overset{L}{\underset{i=1}{\text{∑}}}W_{.i}H_{.j}$, where *L* should be chose to satisfy (*M* + *N*) *L* < *MN*.

In the purpose of finding the appropriate factors *W* and *H*, the commonly used two objective functions are depicted as [18]:

(1) $$E(V\|WH)={\left\|V-WH\right\|}_{F}^{2}=\sum_{i=1}^{M}\sum_{j=1}^{N}{\left(V_{ij}-{\left(WH\right)}_{ij}\right)}^{2}$$

(2) $$D(V\|WH)=\sum_{i=1}^{M}\sum_{j=1}^{N}\left(V_{ij}\log\frac{V_{ij}}{{\left(WH\right)}_{ij}}-V_{ij}+{\left(WH\right)}_{ij}\right)$$

In respect to Equations (1) and (2), ∀*i, a, j* subject to *W_ia_* > 0 and *H_aj_* > 0, *a* is a integer. ‖•‖*_F_* is the Frobenius norm, Equation (1) is called as the Euclid distance while Equation (2) is referred to as K-L divergence function. Note that, finding the approximate solution to *V* ≈ *WH* is considered equal to the optimization of the above mentioned two objective functions.

### 3.2. Accelerated Nonnegative Matrix Factorization (ANMF)

Roughly speaking, the NMF algorithm has high time complexity that results in limited advantages for the overall performance of algorithm, so that the introduction of improved iteration rules to optimize the NMF is extremely crucial to promote the efficiency. In the point of algorithm optimization, NMF is a majorization problem that contains a non-negative constraint. Until now, a wide range of decomposition algorithms have been investigated on the basis of non-negative constraints, such as the multiplicative iteration rules, interactive non-negative least squares, gradient method and projected gradient [29], among which the projected gradient approach is capable of reducing the time complexity of iteration to realize the NMF applications under mass data conditions. In addition, these works are distinguished by meaningful physical significance, effective sparse data, enhanced classification accuracy and striking time decreases. We propose a modified version of projected gradient NMF that will greatly reduce the complexity of iterations; the main idea of the algorithm is listed below.

As we know, the Lee-Seung algorithm continuously updates *H* and *W*, fixing the other, by taking a step in a certain weighted negative gradient direction, namely:

(3) $$H_{ij}\leftarrow H_{ij}-\eta_{ij}{\left[\frac{\partial f}{\partial H}\right]}_{ij}\equiv H_{ij}+\eta_{ij}{\left(W^{T}A-W^{T}WH\right)}_{ij}$$

(4) $$W_{ij}\leftarrow W_{ij}-\varsigma_{ij}{\left[\frac{\partial f}{\partial W}\right]}_{ij}\equiv W_{ij}+\varsigma_{ij}{\left(AH^{T}-WHH^{T}\right)}_{ij}$$

where *η_ij_* and *ζ_ij_* are individual weights for the corresponding gradient elements, which are expressed like follows:

(5) $$\eta_{ij}=\frac{H_{ij}}{{\left(W^{T}WH\right)}_{ij}},\varsigma_{ij}=\frac{W_{ij}}{{\left(WHH^{T}\right)}_{ij}}$$

and then the updating formulas are:

(6) $$H_{ij}\leftarrow H_{ij}\frac{{\left(W^{T}A\right)}_{ij}}{{\left(W^{T}WH\right)}_{ij}},W_{ij}\leftarrow W_{ij}\frac{{\left(AH^{T}\right)}_{ij}}{{\left(WHH^{T}\right)}_{ij}}$$

We notice that the optimal *H* related to a fixed *W* can be obtained, column by column, by independently:

(7) $$\begin{array}{ccc} \min\frac{1}{2}{\left\|Ae_{j}-WHe_{j}\right\|}_{2}^{2} & \text{s.t.} & He_{j}\geq 0 \end{array}$$

where *e_j_* is the *j^th^* column of the *n* × *n* identity matrix. Similarly, we can also acquire the optimal *W* relative to a fixed *H* by solving, row by row:

(8) $$\begin{array}{ccc} \min\frac{1}{2}{\left\|A^{T}e_{i}-HW^{T}e_{i}\right\|}_{2}^{2} & \text{s.t.} & W^{T}e_{i}\geq 0 \end{array}$$

where *e_i_* is the *i^th^* column of the *m* × *m* identity matrix. Actually, both Equations (7) and (8) can be changed into an ordinary form:

(9) $$\begin{array}{ccc} \min\frac{1}{2}{\left\|Ax-b\right\|}_{2}^{2} & \text{s.t.} & x\geq 0 \end{array}$$

where *A* ≥ 0 and *b* ≥ 0. As the variables and given data are all nonnegative, the problem is therefore named the Totally Nonnegative Least Squares (TNNLS) issue.

We propose to revise the algorithm claimed in article [17] by using the same update rule with step-length *α* in [27] to the successive updates in improving the objective functions about the two TNNLS problems mentioned in Equations (7) and (8) As a result, this brings about a modified form of the Lee-Seung algorithm that successively updates the matrix *H* column by column and *W* row by row, with individual step-length *α* and *β* for each column of *H* and each row of *W* respectively. So we try to write the update rule as:

(10) $$H_{ij}\leftarrow H_{ij}+\alpha_{j}\eta_{ij}{\left(W^{T}A-W^{T}WH\right)}_{ij}$$

(11) $$W_{ij}\leftarrow W_{ij}+\beta_{i}\varsigma_{ij}{\left(AH^{T}-WHH^{T}\right)}_{ij}$$

where *η_ij_* and *ζ_ij_* are set equal to some small positive number as described in [27], *α_j_* (*j* = 1,2,…,*n*) and *β_i_* (*i* = 1,2,…,*m*) are step-length parameters can be computed as follows. Let *x* > 0, *q* =*A^T^*(*b* − *Ax*) and *p* = [*x*./(*A^T^Ax*)] ○ *q*, where the symbol “./” means component-wise division and “○” denotes multiplication. Then we introduce variable *ô* ∈ (0, 1):

(12) $$\alpha =\min(\frac{p^{T}q}{p^{T}A^{T}Ap},\tau \max\{\hat{\alpha }:x+\hat{\alpha }p\geq 0\})$$

We can easily obtain the step-length formula of *α_j_* or *β_i_* if (*A, b, x*) is replaced by (*W, Ae_j_, He_j_*) or (*H^T^, A^T^e_i_, W^T^e_i_*), respectively. It is necessary to point out that *q* is the negative gradient of the objective function, and the search direction *p* is a diagonally scaled negative gradient direction. The step-length *α* or *β* is either the minimum of the objective function in the search direction or a *τ*-fraction of the step to the boundary of the nonnegative quadrant.

Learning from article [27] that both quantities, *p^T^q/p^T^A^T^Ap* and *max*{*â* : *x* + *âp* ≥ 0} are greater than 1 in the definition of the step *α*, thereby, we make *α_j_* ≥ 1 and *β_i_* ≥ 1 by treating *τ* sufficiently close to 1. In our experiment, we choose *τ* = 0.99 which practically guarantees that *α* and *β* are always greater than 1.

Obviously, when *α*←1 or *β*←1, update Equations (10) and (11) reduce to updates Equations (3) and (4) In our algorithm, the step-length parameters are allowed to be greater than 1. It is this indicates that for any given (*W, H*), we can get at least the same or greater decrease in the objective function than the algorithm in [27]. Hence, we call the proposed algorithm the Accelerated NMF (ANMF). Besides, the experiments in Section 5.5 will demonstrate that ANMF algorithm is indeed superior to that algorithm by generating better test results, especially when the amount of iterations is not too big.

## 4. The ANMF and NSCT Combined Algorithm

### 4.1. The Selection of Fusion Rules

As we know, approximation of an image belongs to the low-frequency part, while the high-frequency counterpart exhibits detailed features of edge and texture. In this paper, the NSCT method is utilized to separate the high and low components of the source image in the frequency domain, and then the two parts are dealt with different fusion rules according to their features. As a result, the fused image can be more complementary, reliable, clear and better understood.

By and large, the low-pass sub-band coefficients approximate the original image at low-resolution; it generally represents the image contour, but high-frequency details such as edges, region contours are not contained, so we take the ANMF algorithm to determine the low-pass sub-band coefficients which including holistic features of the two source images. The band-pass directional sub-band coefficients embody particular information, edges, lines, and boundaries of region, the main function of which is to obtain as many spatial details as possible. In our paper, a NHM based local self-adaptive fusion method is adopted in band-pass directional sub-band coefficients acquisition phase, by calculating the identical degree of the corresponding neighborhood to determine the selection for band-pass coefficients fusion rules (*i.e.*, regional energy or global weighted).

### 4.2. The Course of Image Fusion

**Figure 2.**
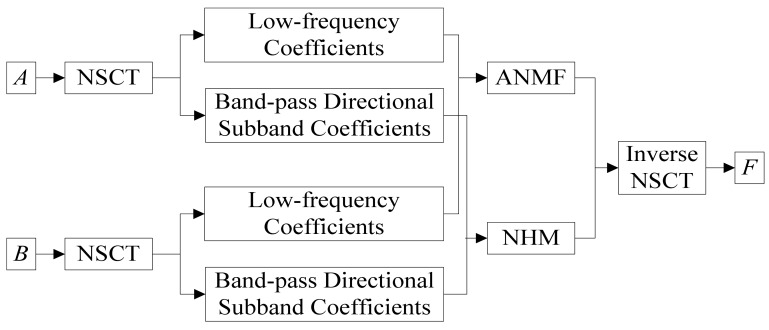
Flowchart of fusion algorithm.

Given that the two source images are *A* and *B*, with the same size, both have been registered, *F* is fused image. The fusion process is shown in Figure 2 and the steps are given as follows (Figure 2):

1. Adopt NSCT to implement the multi-scale and multi-direction decompositions for source images *A* and *B*, and the sub-band coefficients $\left\{C_{i_{0}}^{A}(m,n),C_{i,l}^{A}(m,n)\right\}$, $\left\{C_{i_{0}}^{B}(m,n),C_{i,l}^{B}(m,n)\right\}$ can be obtained.
2. Construct matrix *V* on the basis of low-pass sub-band coefficients $C_{i_{0}}^{A}(m,n)$ and $C_{i_{0}}^{B}(m,n)$ :
  (13) $$V=[v_{A},v_{B}]=\left[\begin{array}{cc} \begin{array}{l} v_{1A}\\ v_{2A}\\ \cdots \\ v_{nA} \end{array} & \begin{array}{l} v_{1B}\\ v_{2B}\\ \cdots \\ v_{nB} \end{array} \end{array}\right]$$
  where *v_A_* and *v_B_* are column vectors consisting of pixels coming from *A* and *B*, respectively, according to principles of row by row. *n* is the number of pixels of source image. We perform the ANMF algorithm described in Section 3.2 on *V*, from which *W* that is actually the low-pass sub-band coefficients of fused image *F* is separated. We set maximum iteration number as 1,000 with *τ* = 0.99.
  The fusion rule NHM is applied to band-pass directional sub-band coefficients $C_{i,l}^{A}(m,n)$, $C_{i,l}^{B}(m,n)$ of source images *A, B*. The NHM is calculated as:
  (14) $$NHM_{i,j}(m,n)\frac{2•\{\underset{\left(k,j)\in N_{i,j}(m,n\right)}{\text{∑}}\left|C_{i,j}^{A}(m,n)|•|C_{i,j}^{B}(m,n)\right|\}}{E_{i,j}^{A}(m,n)+E_{i,j}^{B}(m,n)}$$
  where *E_i,l_*(*m, n*) is regarded as the neighborhood energy under resolution of *2^l^* in direction *i, N_i,l_* (*m, n*) is the 3 × 3 neighborhood centers at point (*m, n*). In fact, NHM quantifies the identical degree of corresponding neighborhoods for two images, the higher the identical degree is, the greater the NHM value should be. Because 0 ≤ *NHM_i,l_*(*m, n*) ≤ 1, we define a threshold *T*; generally we have it that 0.5 < *T* < 1. As the quality of fusion image is partly influenced by *T* (see Table 1), we take two factors into consideration [*i.e.*, when *T* =0.75 the SD (Standard Deviation) and AG (Average Gradient) are better], so the threshold is given as *T* = 0.75. The fusion rule of band-pass directional sub-band coefficients is expressed as:
  when *NHM_i,l_* (*m, n*) < *T*:
  $$\left\{ \begin{array}{l} C_{i,l}^{F}(m,n)=C_{i,l}^{A}(m,n)\;\text{if}\;E_{i,l}^{A}(m,n)\geq E_{i,l}^{B}(m,n)\\ C_{i,l}^{F}(m,n)=C_{i,l}^{B}(m,n)\;\text{if}\;E_{i,l}^{A}(m,n)<E_{i,l}^{B}(m,n) \end{array}\right.$$
  when *NHM_i,l_* (*m, n*) ≥ *T*:
  $$C_{i,l}^{F}(m,n)=NHM_{i,l}(m,n)•\max(C_{i,l}^{F}(m,n),C_{i,l}^{A}(m,n))+(1-NHM_{i,l}(m,n))•\min(C_{i,l}^{F}(m,n),C_{i,l}^{B}(m,n))$$
3. Perform inverse NSCT transform on the fusion coefficients of *F* obtained from step (2) and get the ultimate fusion image *F*.

## 5. Experiments and Analysis

### 5.1. Experimental Conditions and Quantified Evaluation Indexes

To verify the effectiveness of the proposed algorithm, three groups of images are used under the MATLAB 7.1 platform in this Section. All source images must be registered and with 256 gray levels. By comparison with the five typical algorithms below: NSCT-based method (M1), NMF-based method (classic NMF, M2), weighted NMF-based method (M3), PCA and wavelet, we can learn more about the one presented in our paper.

It may be possible to evaluate the image fusion subjectively, but subjective evaluation is likely affected by the biases of different observers, psychological status and even mental states. Consequently, it is absolutely necessary to establish a set of objective criteria for quantitative evaluation. In this paper, we select the Information Entropy (IE), Standard Deviation (SD), Average Gradient (AG), Peak Signal to Noise Ratio (PSNR), Q index [30], Mutual Information (MI), and Expanded Spectral Angle Mapper (ESAM) [31] as our evaluation metrics. IE is one of the most important evaluation indexes, whose value directly reflects the amount of information in the image. The larger the IE is the more information is contained in a fused image. SD indicates the deviation degree between the gray values of pixels and the average of the fused image. In a sense, the fusion effect is in direct proportion to the value of the SD. AG is capable of expressing the definition extent of the fused image, the definition extent will be better with an increasing AG value. PSNR is the ratio between the maximum possible power of a signal and the power of corrupting noise. The larger the PSNR is, the better is the image. MI is a quantity that measures the mutual dependence of the two random variables, so a better fusion effect makes for a bigger MI. Q index measures the amount of edge information “transferred” from source images to the fused one to give an estimation of the performance of the fusion algorithm. Here, larger Q value means better algorithm performance. ESAM is an especially informative metric in terms of measuring how close the pixel values of the two images are and we take the AE (average ESAM) as an overall quality index for measuring the difference between the two source images and the fused one. The higher the AE, the less the similarity of two images will be. The AE is computed using a sliding window approach, in this work, sliding windows with a size of 16 × 16, 32 × 32, and 64 × 64 are used.

### 5.2. Multi-Focus Image Fusion

**Figure 3.**
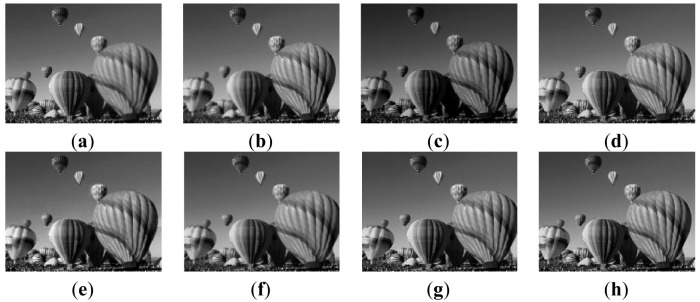
Multi-focus source images and fusion results. (**a**) Left-focused image; (**b**) Right-focused image; (**c**) Fused image based on M1; (**d**) Fused image based on M2; (**e**) Fused image based on M3; (**f**) Fused image based on PCA; (**g**) Fused image based on wavelet; (**h**) Fused image based on our method.

A pair of “Balloon” images are chose to be source images, both are 200 by 160 in size. As can be seen from Figure 3(a), the left side of the image is in focus while the other side is out of focus. The opposite phenomenon emerges in Figure 3(b). Six variant approaches, M1–M3, PCA, wavelet (bi97), and our method, are applied to test the fusion performance. Figure 3c–h) show the simulated results.

From an intuitive point of view, the M1method produces a poor intensity that makes Figure 3(c) somewhat dim. On the contrary, the other five algorithms generate better performance in this aspect, but artifacts located in the middle right of Figure 3(e) can be found. Compared with the M2 and M3 methods, although the definition of the bottom left region in our method is slightly lower than that of the two algorithms, the holistic presentation is superior to the two. As for PCA and wavelet, the similar visual effects as Figure 3(h) are obtained, except the middle bottom balloon in Figure 3(f) is slightly blurred. Statistic results in Tables 2 and 3 verify the above visual effects further.

Table 2 illustrates that the proposed method has advantages over most of other algorithms since all the criteria, in both protection of image details and fusion of image information, are superior to that of M1–M3 and PCA. Of them, the indexes IE, SD, AG, PSNR of our method exceed those of M1, M2, M3 and PCA by 3.1%, 1.3%, 1.5% and 0.8% (for IE), 6.0%, 2.7%, 2.1% and 1.1% (for SD), 0.6%, 3.3%, 0.6% and 0.3 (for AG), 5.3%, 2.6%, 1.8% and 1.3% (for PSNR), respectively. These four basic indices indicate that our method provides a better visual effect. As for index Q, the 0.9844 value of our method means the best fusion algorithm performance when compared to values of the former four algorithms. In MI, our method is also the best, being superior to that of M1 by 19%; the latter is in effect the worst one. As for wavelet, four of six metrics are slightly inferior to ours while two of six metrics are inferior to ours. From Table 3, it can be found that our method has the lowest AE (AE_aF_, AE_bF_ denote similarity between source image (a) and the fused one; (b) and the fused one, respectively) followed by wavelet, M3, PCA, M2, and method M1 has the highest AE. Therefore, in terms of transferring details, the performances of our method, wavelet, M3, PCA, M2, and M1 decrease.

### 5.3. Medical Image Fusion

**Figure 4.**
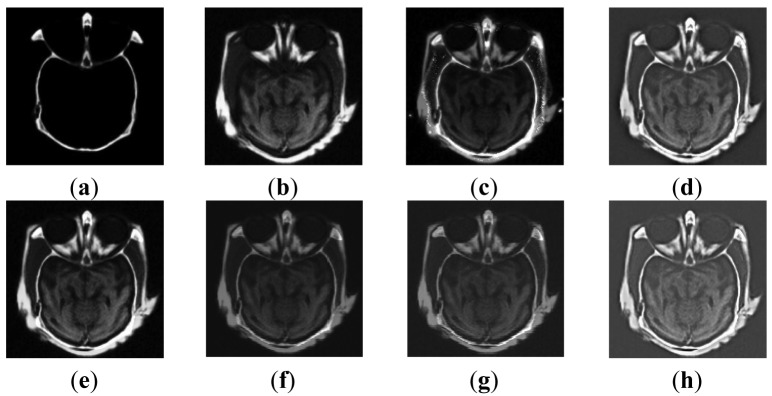
Medical source images and fusion results. (**a**) CT image; (**b**) MRI image; (**c**) Fused image based on M1; (**d**) Fused image based on M2; (**e**) Fused image based on M3; (**f**) Fused image based on PCA; (**g**) Fused image based on wavelet; (**h**) Fused image based on our method.

Figure 4(a,b) are medical CT and MRI images whose sizes are 256 by 256. Six different methods, including our proposed one, are adopted to evaluate the fusion performance, and the simulated results are shown in Figure 4(c–h).

From Figure 4, images based on methods M2 and M3 are not fused well enough for the information in the MRI source image is not fully described yet. Although the external contour of M1 is clear, the overall effect is poor, which is confirmed by the low brightness of the image and the appearance of some undesirable artifacts observed on both sides of the cheek. Oppositely, PCA, wavelet and our methods not only produce distinct outlines and rationally control the brightness level, but also preserve and enhance image detailed information well. Related evaluations are recorded in Tables 4 and 5.

As revealed in Table 4, the proposed method is nearly the best based on the fact that the metrics of IE, SD, AG, PSNR of Figure 4(h) are all greater than that of the former four algorithms (percentages are not listed). The IE value of M1 is the lowest, which is precisely in accord with the image. Our method possesses an AG index of 29.209 which implies the image is clearer than images based on other approaches. In PSNR and SD, our method performs well, being second only to the wavelet approach, and the SD of M1 beats that of M2 and M3. As to Q index and MI, our method takes the second place in MI and the first place in Q, which indicates that the details and edges from source images are well inherited. These details and edges are extremely important for medical diagnosis. Like in experiment 1, our method achieves the lowest values both in AE_aF_ and AE_bF_, and that of wavelet, M3, PCA, M2 and M1 arrange in ascending order.

### 5.4. Visible and Infrared Image Fusion

**Figure 5.**
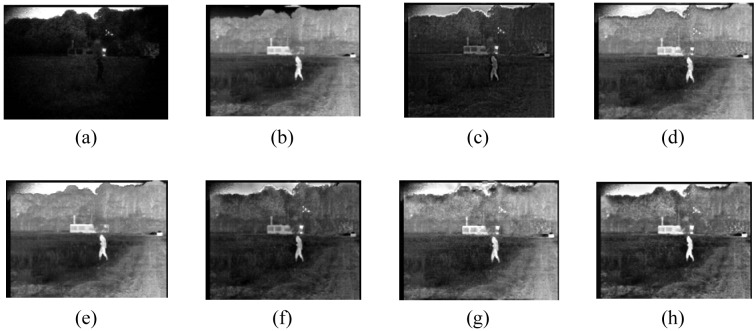
Visible and infrared source images and fusion results. (**a**) Visible band image; (**b**) Infrared band image; (**c**) Fused image based on M1; (**d**) Fused image based on M2; (**e**) Fused image based on M3; (**f**) Fused image based on PCA; (**g**) Fused image based on wavelet; (**h**) Fused image based on our method.

A group of registered visible and infrared images with a size of 360 by 240 showing a person walking in front of a house are labeled as Figure 5(a,b).

Of these, Figure 5(a) has a clear background, but infrared thermal sources cannot be detected. Conversely, Figure 5(b) highlights the person and house but its ability to render other surroundings is weak. Effective fusions are achieved by the six methods. After concrete analysis on the six fused images, we draw the following conclusions: we can find that the image based on method M1 is the worst in overall effect, especially a dark area around the person, which is partly caused by the significant differences between two source images. Method M2 produces more smooth details than M1, as a case in point, the road on the right side of the image and the grass on the other side can easily be recognized for the enhancement of intensity. Approximate effects displayed in Figure 5(e–h) are achieved by using M3, PCA, wavelet and our method, from which we can easily distinguish most parts of the scene except the lighting beside the house in Figure 5(e) that can hardly be observed. It is difficult to judge the performances of the latter four methods through visual observation in case of the concrete data are not provided by Table 6.

In so far as IE, AG, and PSNR are concerned, the proposed technique is evidently better than the former four ones. Specially, the value of our method exceeds them by 1.6%, 4.9%, and 0.7% while the SD is slightly smaller when compared with M3. In index Q, the optimal value is obtained on the basis of the wavelet approach, while that of M1 holds the final place. As for MI, our method still ranks the first place in Table 6. Analogous effects are achieved in Table 7, statistics show that the similarities between visible light, infrared and fused images generated by our method are the best in that both AE_aF_ and AE_bF_ are the smallest.

### 5.5. Numerical Experiment on ANMF

**Figure 6.**
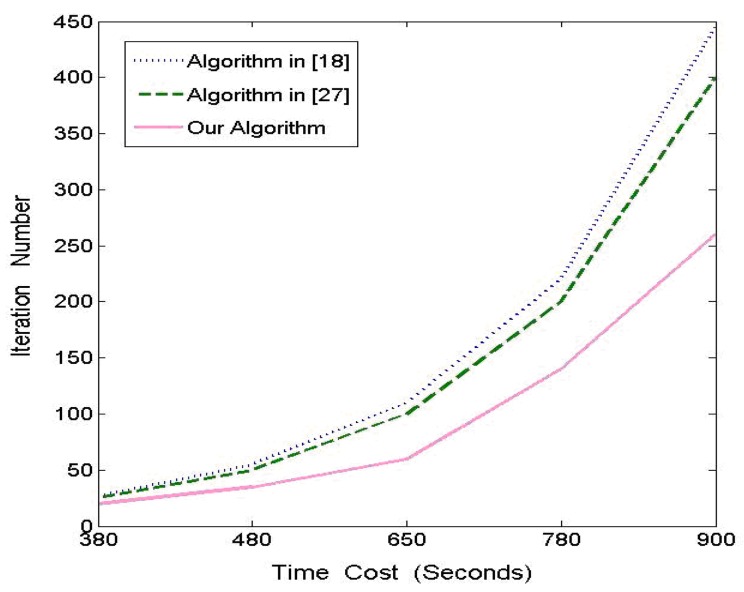
Numerical comparison between three algorithms.

In this section, we compare the performance of ANMF with that of algorithms presented in article [27] and [18] in order to prove its advantages. The algorithms are implemented in Matlab and applied to the Equinox face database [32]. The contrast experiments are conducted four times, where *p* is as described in Section 3.2 and *n* denotes for the number of images chosen from the face database. The Y axis of Figure 6 represents the number of iterations repeated by the three algorithms and the X axis is the time consumption scale. We choose one group of these experiments and demonstrate the results in Figure 6 with *p* = 100 and *n* = 1,000, in which algorithm in [18] is first performed for a given number of iterations and record the time elapsed and then run algorithm in [27] and our algorithm until the time consumed is equivalent to that of the former, respectively. We note that our algorithm offers improvements in all given time points, however, the relative improvement percentage of our method over other two algorithms goes down when the number of iterations increases. Actually, the performance of our method increases about 36.8%, 26.4%, 15.7%, 12.6%, 7.5% and 37.9%, 29.6%, 19.4%, 17.8%, 12.6%, respectively, when comparing with the algorithms in [27] and [18] at each of five time points. In other words, our method converges faster, especially at the early stages, but the percentage tends to decline, which implies that this attribute is merely useful for real-time applications without very large-scale data sets.

### 5.6. Discussion

Image fusion with different models and numerical tests are conducted in our experiments, where the above four experiments indicate that the proposed method has a notable superiority in image fusion performance over the four other techniques examined (see Sections 5.2–5.4), and has better iteration efficiency (see Section 5.5). We observed that images based on wavelet and our proposed methods enjoy the best visual effect, and then the PCA, M3, M2, and M1 are the worst. In addition to visual inspection, quantitative analysis is also conducted to verify the validity of our algorithm from the angles of information amount, statistical features, gradient, signal to noise ratio, edge preservation, information theory and similarity of structure. The values in these metrics prove that the experiments achieve the desired objective.

## 6. Conclusions

In this paper, we have presented a technique for image fusion based on the NSCT and ANMF model. The accelerated NMF method modifies the traditional update rules of *W* and *H*, which achieves better effect by adopting the theory of matrix decomposition. The current approaches on the basis of NMF usually need more iterations to converge when compared to the proposed method, but the same or better results can be attained by our technique via less iterations. The results of simulation experiments show that the proposed algorithm can not only reduce computational complexities, but achieve better or equal performances when compared with other mentioned techniques both from the visual and statistical standpoints. The optimization for our method will be the next step in order to apply it in large scale data sets.

## Figures and Tables

**Table 1.** The tradeoff selection for *T*.

*T*	SD	AG	*T*	SD	AG
0.55	30.478	8.3784	0.75	30.539	8.5109
0.6	30.664	8.4322	0.8	30.541	8.4376
0.65	30.412	8.4509	0.9	30.629	8.4415
0.7	30.456	8.5322	0.95	30.376	8.2018

**Table 2.** Comparison of the fusion methods for multi-focus images.

	**M1**	**M2**	**M3**	**PCA**	**Wavelet**	**Proposed method**
IE	7.3276	7.4594	7.4486	7.4937	7.5982	7.5608
SD	28.705	29.728	29.934	30.206	31.127	30.539
AG	8.4581	8.2395	8.4595	8.4853	8.5014	8.5109
PSNR(dB)	35.236	36.246	36.539	36.746	37.533	37.224
Q Index	0.9579	0.9723	0.9706	0.9812	0.9901	0.9844
MI	3.4132	3.5268	3.9801	4.0538	4.1257	4.2578

**Table 3.** ESAM values between multi-focus and fused images.

	**M1**	**M2**	**PCA**	**M3**	**Wavelet**	**Proposed method**
AE_aF_16 × 16	20.37	19.96	19.89	19.82	19.27	18.96
AE_aF_32 × 32	19.85	19.32	19.29	19.24	18.95	18.42
AE_aF_64 × 64	19.06	18.62	18.53	18.42	18.13	17.95
AE_bF_16 × 16	20.08	19.43	19.38	19.35	18.87	18.54
AE_bF_32 × 32	19.62	18.88	18.81	18.76	18.11	17.96
AE_bF_64 × 64	18.98	18.27	18.15	18.03	17.66	17.38

**Table 4.** Comparison of the fusion methods for medical images.

	**M1**	**M2**	**M3**	**PCA**	**Wavelet**	**Proposed method**
IE	5.4466	5.7628	5.7519	5.8875	6.1022	6.0641
SD	29.207	27.768	27.883	28.549	31.836	31.628
AG	20.361	26.583	25.194	27.358	28.573	29.209
PSNR(dB)	36.842	37.238	37.428	37.853	38.737	38.458
Q Index	0.9607	0.9695	0.9714	0.9821	0.9874	0.9835
MI	4.0528	4.3726	4.3942	4.5522	4.8736	5.0837

**Table 5.** ESAM values between CT, MRI and fused images.

	**M1**	**M2**	**PCA**	**M3**	**Wavelet**	**Proposed method**
AE_aF_16 × 16	18.45	18.09	17.83	17.64	17.33	17.04
AE_aF_32 × 32	18.13	17.67	17.32	17.08	16.79	16.58
AE_aF_64 × 64	17.74	17.22	16.95	16.82	16.57	16.12
AE_bF_16 × 16	18.39	18.12	17.79	17.53	17.38	17.11
AE_bF_32 × 32	18.08	17.74	17.21	17.09	16.91	16.62
AE_bF_64 × 64	17.76	17.36	17.05	16.85	16.34	16.17

**Table 6.** Comparison of the fusion methods for visible and infrared images.

	**M1**	**M2**	**M3**	**PCA**	**Wavelet**	**Proposed method**
IE	6.2103	6.3278	6.6812	6.7216	6.8051	6.7962
SD	23.876	22.638	25.041	24.865	25.137	25.029
AG	3.2746	3.0833	3.3695	3.4276	3.5234	3.5428
PSNR(dB)	37.093	38.267	38.727	38.971	39.765	39.021
Q Index	0.9761	0.9784	0.9812	0.9836	0.9956	0.9903
MI	3.8257	4.2619	4.3128	4.5595	4.6392	4.7156

**Table 7.** ESAM values between visible, infrared and fused images.

	**M1**	**M2**	**PCA**	**M3**	**Wavelet**	**Proposed method**
AE_aF_16 × 16	22.53	22.17	21.88	21.69	21.14	21.03
AE_aF_32 × 32	22.14	21.84	21.65	21.13	20.82	20.56
AE_aF_64 × 64	21.75	21.36	20.83	20.52	20.06	19.94
AE_bF_16 × 16	22.44	22.13	21.76	21.38	21.03	20.87
AE_bF_32 × 32	22.08	21.22	20.93	20.69	20.47	20.15
AE_bF_64 × 64	21.69	20.87	20.55	20.07	19.89	19.68
